# Spatially Adaptive Convolutional Networks with Coordinate-Conditioned Layers

**DOI:** 10.1145/3678717.3691253

**Published:** 2024-11-22

**Authors:** Heather Baier, Dan Runfola

**Affiliations:** College of William and Mary, Williamsburg, Virginia; College of William and Mary, Williamsburg, Virginia

**Keywords:** Adaptive Weights, Spatial Autocorrelation, Convolutional Layers, Socioeconomic

## Abstract

In this study, we present a convolutional neural network (CNN) architecture, GeoConv, designed to improve the accuracy and adaptability of deep learning models using satellite imagery. Traditional CNNs, such as ResNet18, employ fixed-weight convolutional layers - i.e., layers that leverage the same set of weights for each input observation. However, these models can struggle to capture context-specific features inherent in satellite images, which may vary significantly across different geographic regions. To address this challenge, the GeoConv model utilizes dynamic weights that adapt based on the input image coordinates, allowing the model to tailor its feature extraction process to the unique characteristics of different geographic regions. Through experiments, we illustrate the utility of this approach in a case study which leverages satellite imagery to estimate household wealth across 11 countries, with GeoConv explaining an additional 10.12% of the variance in the data compared to a ResNet18 model. These results underscore the importance of incorporating spatially adaptive mechanisms in handling the variability present in satellite imagery. Code is available at: https://github.com/heatherbaier/geoconv

## 1 INTRODUCTION AND RELATED WORK

Convolutional Neural Networks (CNNs) have achieved significant success across various visual recognition tasks [10, 11]. In most applications, these networks employ fixed-weight convolutional layers, applying the same weights consistently across all inputs [12]. While this approach is effective for tasks with consistent visual features, it is challenged by the spatial variability inherent in satellite imagery, where features can vary significantly across geographic contexts [19].

We study this challenge in the context of predicting socioeconomic indicators - such as household wealth - from satellite imagery, a recent approach being utilized in the context of international development and the social sciences more broadly [8, 15, 16]. In this context, the generalizability of fixed-weight CNNs across diverse geographic regions is limited [4, 8], as the relationship between visual features and wealth can differ markedly between urban and rural areas [5]. Scholars have explored various techniques, but broad-scope estimation across multiple countries remains a challenge [3, 14].

Several models address spatial heterogeneity in deep learning. The Spatial Ensemble Learning (SEL) framework clusters data into zones and trains local classifiers to manage class ambiguity and spatial dependencies [9]. The Spatial Transformation And modeRation (STAR) model dynamically partitions the spatial domain, introducing a hierarchical structure with a spatial moderator for broader adaptability [17]. The Spatial Variability Aware Neural Network (SVANN) modifies either its architecture or weights based on location, enabling tailored responses to geographic variations [6].

Here, we build on these frameworks and present a model which explicitly leverages geographic coordinate-based conditioning to vary weights across individual inputs using hypernetworks. Hypernetworks have recently emerged as a tool to enhance model predictions by dynamically incorporating metadata into model weights [7]. In spatial contexts, hypernetworks have been used to generate weights based on factors like image capture time and sensor-specific spectral wavelengths [13, 18].

In contrast to existing spatial variability aware networks, which tend to focus on region-based model adaptation, GeoConv directly integrates spatial metadata into hypernetwork layers for more flexible, fine-grained spatial adjustments. We demonstrate that GeoConv significantly improves the generalization of wealth estimation from satellite imagery across multiple countries by tailoring its feature extraction to the specific characteristics of each region. Our approach addresses the limitations of fixed-weight CNNs in spatially diverse contexts and provides a flexible, scalable solution for satellite-based socioeconomic analysis.

## 2 METHODOLOGY

GeoConv builds on the ResNet18 framework by integrating adaptive convolutional layers that dynamically adjust based on the geographic coordinates of the input images. The architecture begins with an initial GeoConv 3×3 layer, followed by max pooling, three basic blocks, an adaptive block, and finally average pooling leading to a linear classification layer. This integration allows the network to tailor its feature extraction process to the specific characteristics of different regions, improving the model’s ability to capture and interpret the complex spatial patterns inherent in satellite imagery.

The implementation of coordinate-conditioned convolutional layers in GeoConv involves four main steps:

### (1) Coordinate Projection:

The input image’s 2D coordinate vector, representing the latitude and longitude, is initially projected into a higher-dimensional space to enable more complex mappings. Specifically, this is done using a linear layer that transforms the 2D coordinates into a 128-dimensional vector. The transformation is governed by the equation:

$$\upsilon_{1}=\text{ReLU}(W_{1}c+b_{1})$$

where $W_{1}\in {\mathbb{R}}^{128\times 2}$ represents the weight matrix, and $b_{1}\in {\mathbb{R}}^{128}$ is the bias vector. The ReLU activation function ensures that the projection captures non-linear relationships between the coordinates and the subsequent convolutional weights.

### (2) Feature Mapping:

The 128-dimensional vector $\upsilon_{1}$ generated in the previous step is then further mapped to match the number of weights required for the convolutional filters in the network. For example, if we target a convolutional layer with 64 input channels, 64 output channels, and a 3×3 kernel, the resulting number of parameters would be 36,864. This is achieved through a second linear layer that takes the 128-dimensional vector and outputs a vector $\upsilon_{2}$ with the required number of elements:

$$\upsilon_{2}=\text{Sigmoid}\left(W_{2}\upsilon_{1}+b_{2}\right)$$

Here, $W_{2}\in {\mathbb{R}}^{d\times 128}$ and $b_{2}\in {\mathbb{R}}^{d}$ represent the weight matrix and bias vector of the second linear layer, respectively, where d is the dimensionality needed to reshape $\upsilon_{2}$ into the convolutional kernel.

### (3) Kernel Shaping:

The output vector $\upsilon_{2}$ is then reshaped to form the convolutional kernel weights. The reshaping process converts the 1-dimensional output vector into a 4-dimensional tensor $W^{\prime }$, which represents the dynamically generated weights for the convolutional operation:

$$W^{\prime }=\text{reshape}\left(\upsilon_{2},\left[C_{out,}C_{in,}K,K\right]\right)$$

In this context, $C_{out}$ denotes the number of output channels, $C_{in}$ denotes the number of input channels, and K represents the kernel size (e.g., 3×3).

### (4) Convolution Operation:

Finally, the reshaped tensor $W^{\prime }$ is used as the convolutional kernel in the network’s layers. The adaptive nature of these weights allows each convolutional layer to respond specifically to the geographic features of the input image, enhancing the model’s ability to extract meaningful patterns that are relevant to the spatial context.

By dynamically adjusting the convolutional kernel weights based on the input image’s geographic coordinates, GeoConv seeks to improve the network’s ability to delineate features pertinent to different geographic regions. This approach is particularly advantageous for tasks involving satellite imagery, where the spatial variability of features can be substantial.

### 2.1 Layer Placement Strategy

In the GeoConv architecture, we integrate adaptive convolutional layers into a standard ResNet18 model. Specifically, we replace the first convolutional layer and add adaptive layers before the residual connections in the final convolutional block. These adaptive layers use weights computed by a hypernetwork, allowing the model to fine-tune its convolutional filters based on geographic metadata (in our experiments, latitude and longitude). This enables targeted feature extraction relevant to the specific geographic context—for example, emphasizing agricultural features in rural areas or infrastructure-related features in urban regions. The remaining layers follow the standard ResNet18 architecture.

## 3 EXPERIMENTS

### 3.1 Data

#### 3.1.1 USAID Demographic and Health Survey.

We utilize data from the USAID Demographic and Health Surveys (DHS) [1], focusing on the Wealth Index, a composite measure of household socioeconomic status derived from principal component analysis (PCA) of assets and housing characteristics. Wealth scores, standardized and divided into quintiles, are mean-normalized per country as shown in Equation 1:

(1) $$x_{\text{norm}}=\frac{x-\mu }{\max\left(x\right)-\min\left(x\right)}$$

where

- $x_{\text{norm}}$: The mean-normalized value of the data point *x*.
- x: An individual data point within the dataset.
- μ: The mean of all the data points in the dataset.
- $\max\left(x\right)$: The maximum value in the dataset.
- $\min\left(x\right)$: The minimum value in the dataset.

The presented analysis includes information from 11 countries (95,579 data points) in Western Africa.

#### 3.1.2 Planet Satellite Imagery.

We used the 2023 Q3 global composite Planet Basemaps [2] with 3m resolution, clipped to a 0.08km buffer around each DHS point, resulting in 95,579 imagery tiles labeled with the corresponding mean-normalized Wealth Index value.

### 3.2 Implementation Details

All models are trained using PyTorch. We utilize an L1 loss function, an Adam optimizer, and train each model for 200 epochs utilizing 3-kfold cross-validation. Each image has three RGB channels and is cropped to an input size of 224 × 224 × 3. GeoConv utilizes a learning rate of 0.00005, while the other models we contrast to utilize a learning rate of 0.00001. Learning rates were selected after a grid search of hyperparameters.

### 3.3 Training Procedure

We trained the GeoConv model using a single image per training pass, replacing the batch normalization layers with InstanceNorm layers to accommodate the batch size of 1. This approach allows the model to adjust weights based on the metadata of each individual image. To maintain training efficiency, we employed gradient accumulation over 64 images, updating the model parameters after each accumulation and resetting the gradients.

### 3.4 Evaluation Metrics

The performance of each model was evaluated using two metrics: the ${\text{r}}^{2}$ score measures the proportion of variance in the dependent variable predictable from the independent variable(s), and the Mean Absolute Error (MAE) quantifies the average magnitude of errors in a set of predictions, regardless of their direction.

### 3.5 Baseline Models for Comparison

In addition to the GeoConv model, we provide three baseline models to contrast our findings to:

- **ResNet18**: A ResNet18 model trained by aggregating data from all source countries.
- **Spatial-Embed (SEM)**: A ResNet18 architecture with a 2-layer MLP that projects the coordinates of every image into latent space using 64 and 128 element linear layers. The 128-element output of the coordinate projector and the 512 element output from the final convoluional block of the ResNet18 are concatenated into a 640 element vector that is then fed through the final fully connected layer for a wealth prediction.
- **Dynamic Fully Connected Model (DFC)**: In DFC, a hypernetwork is employed to generate the weights of the final fully connected layer of a ResNet18. The hypernetwork takes as input the coordinates of the input image and processes them through 2 linear layers within the hypernetwork, resulting in the dynamic generation of the weights for the final fully connected layer.

These models are contrasted in our discussion to explore the degree to which spatial metadata can improve the estimation of household wealth from satellite images.

## 4 RESULTS

Table 1 presents a comparison of our method and each baseline method based on their mean average error and ${\text{r}}^{2}$ values. Values are averages across 3 folds. ResNet18 is the lowest performing model, achieving an ${\text{r}}^{2}$ accuracy of 0.6027 with and an MAE of 0.0858. SEM shows a slight improvement with an ${\text{r}}^{2}$ accuracy 0.6122 and an MAE of 0.084. DFC further improves performance with an accuracy of 0.6424 and an MAE of 0.0793. Finally, GeoConv outperforms the other methods with an accuracy of 0.7039 and the lowest MAE of 0.0678.

## 5 DISCUSSION

Incorporating spatial metadata consistently improves performance compared to a standard ResNet18 model without spatial context. Among the methods tested, SEM, which directly concatenates spatial information into the fully connected layer, shows a slight improvement over ResNet18 but is outperformed by both DFC and GeoConv. DFC, which conditions the fully connected layer based on geographic coordinates, surpasses SEM but lacks the ability to dynamically extract features. GeoConv, on the other hand, demonstrates superior performance across all baselines by conditioning feature extraction on geographic context, allowing it to adapt more effectively to diverse spatial patterns.

**Figure 1:**
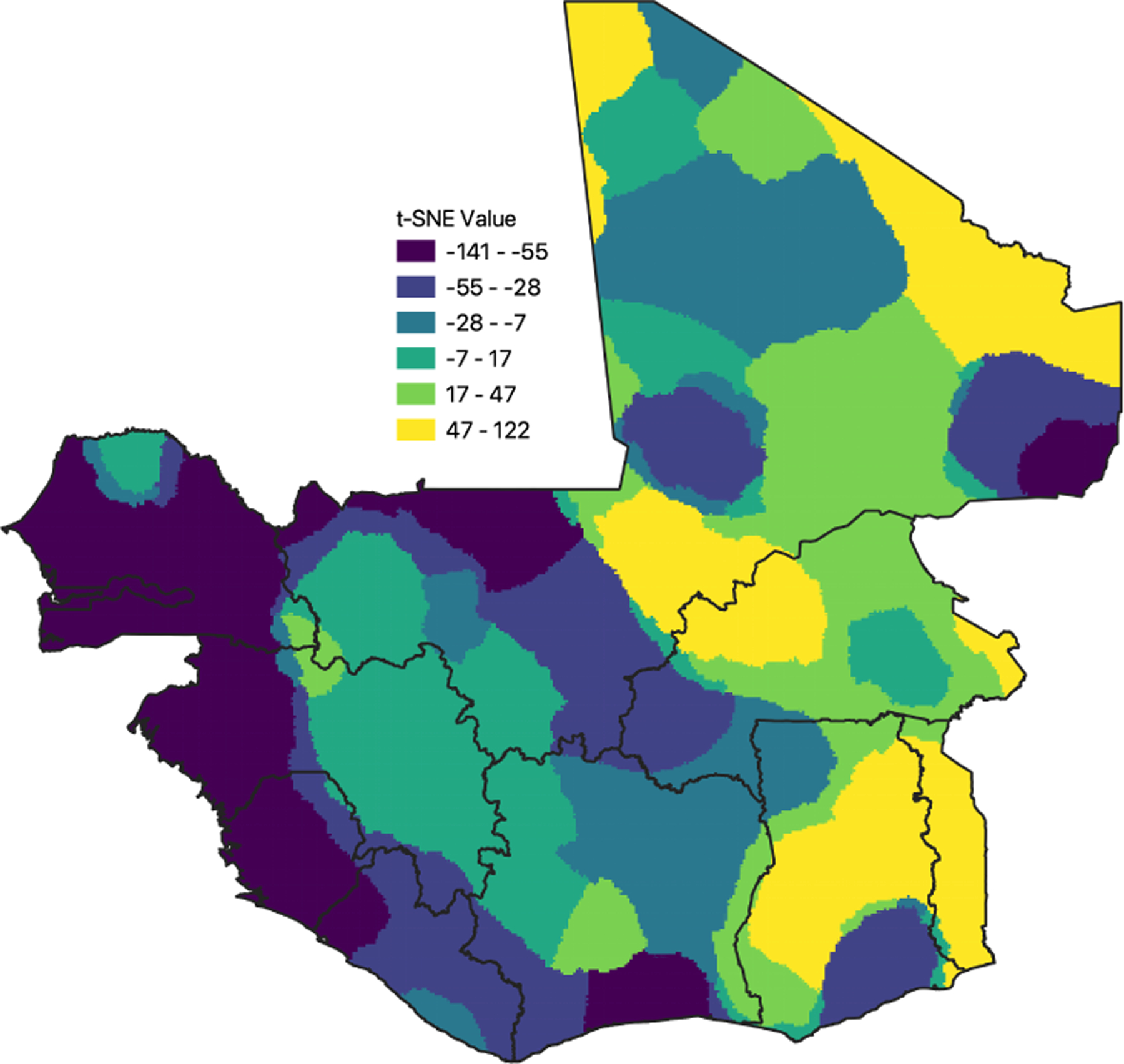
t-SNE of predicted model weights

To understand the geographic variation in the model’s learned features, we analyzed the spatial clustering of convolutional weights predicted by the GeoConv model. Using t-SNE, we visualized these weights, revealing distinct clusters across different regions, indicating that GeoConv captures region-specific relationships between features and socioeconomic outcomes. This spatial clustering was further confirmed by a strong positive spatial autocorrelation (I = 0.99976, z = 521.57; see figure 1), suggesting that similar feature patterns are geographically clustered within the study area.

We also explored the relationship between predicted weights and land cover types, hypothesizing that GeoConv’s dynamic weights might vary across different landscapes. A Kruskal-Wallis test, as shown in table 2, confirmed significant differences in t-SNE values among various land cover types, indicating that the model adapts its feature extraction to the specific characteristics of different environments.

**Figure 2:**
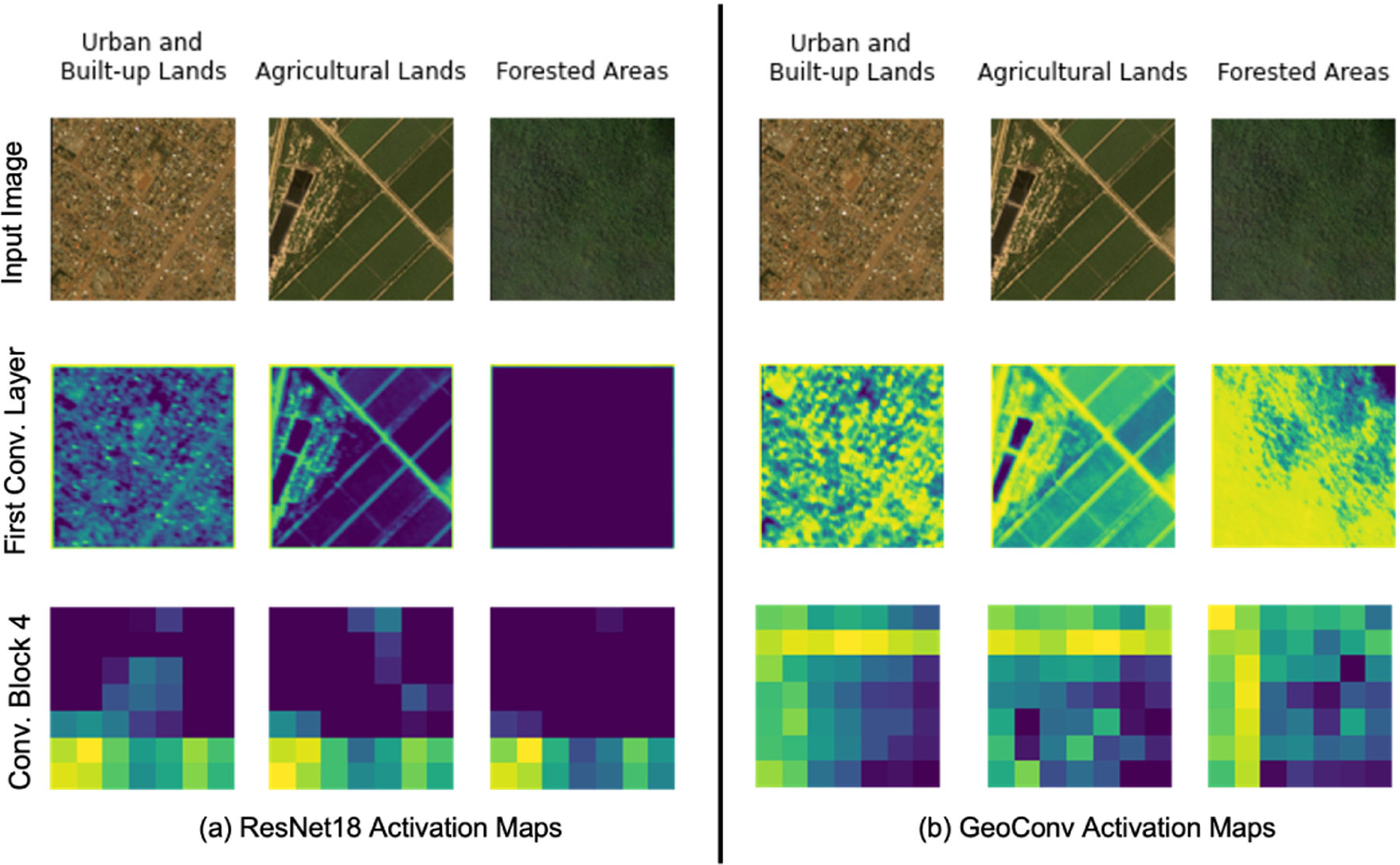
Activation Maps

Finally, as shown in Figure 2, we compared activation maps from the GeoConv and ResNet18 models, observing that GeoConv activates a higher number of neurons across various layers, capturing more detailed spatial features. This increased neuron activation in GeoConv suggests a superior ability to extract and represent fine-grained geospatial information, leading to more accurate and context-aware predictions.

## 6 LIMITATIONS & FUTURE DIRECTIONS

Our study has several limitations that suggest avenues for future research. First, we only tested the GeoConv approach using ResNet18, and its generalizability to other architectures like ResNet50, VGG16, Inception, and Transformer-based models remains uncertain. Evaluating GeoConv across a broader range of architectures would help establish its robustness and versatility. Additionally, our experiments were geographically limited to African datasets, restricting the generalizability of our findings. Expanding the study to include datasets from diverse regions could provide insights into the model’s global applicability. Finally, GeoConv significantly increases the number of parameters compared to ResNet18, resulting in larger model sizes and longer training times. Future work could explore optimizing the architecture to reduce parameters while maintaining performance, improving its efficiency for broader use.

## 7 CONCLUSION

In this piece, we tested the value of introducing dynamic weights into a convolutional model, allowing filter weights to vary based on the latitude and longitude metadata of satellite images. Using a dataset of 95,579 household survey points across 11 African nations, we estimated household wealth with approximately 3-meter resolution satellite imagery. We found that deep learning models without spatial metadata could achieve a regressive accuracy of ${\text{r}}^{2}$ of 0.6027, models with spatial information but no adaptive layers could achieve up to ${\text{r}}^{2}=0.6424$, and the GeoConv approach with adaptive layers achieved ${\text{r}}^{2}=0.7039$. These results highlight the value of introducing adaptive weights into convolutional neural network architectures, specifically in the context of spatial data.

## Supplementary Material

Code

## Figures and Tables

**Table 1:** Modeling Results

Model	${\text{r}}^{\text{2}}$	MAE
ResNet18	0.6027	0.0858
SEM	0.6122	0.084
DFC	0.6424	0.0793

**GeoConv**	**0.7039**	**0.0678**

**Table 2:** Median t-SNE value of each land cover class

Land Cover Type	Median t-SNE Value
Agricultural Lands	−30.86
Forested Areas	22.17
Grasslands and Wetlands	−16.52
Shrublands and Savannas	19.02
Urban and Built-up Lands	−30.28
Water Bodies and Non-Vegetated Lands	−8.29

## CCS CONCEPTS

- Computing methodologies → Neural networks
- Applied computing → Law, social and behavioral sciences
